# LAD1 promotes malignant progression by diminishing ubiquitin-dependent degradation of vimentin in gastric cancer

**DOI:** 10.1186/s12967-023-04401-2

**Published:** 2023-09-17

**Authors:** Yingming Jiang, Yanchun Feng, Jintuan Huang, Zhenze Huang, Rongchang Tan, Tuoyang Li, Zijian Chen, Xiaocheng Tang, Jun Qiu, Chujun Li, Hao Chen, Zuli Yang

**Affiliations:** 1 Department of Gastrointestinal Endoscopy, Department of General Surgery, The Sixth Affiliated Hospital, Sun Yat-Sen University, 510655 Guangzhou, China; 2 Department of Gastric Surgery Section 2, Department of General Surgery, The Sixth Affiliated Hospital, Sun Yat-Sen University, 510655 Guangzhou, China; 3 Guangdong Provincial Key Laboratory of Colorectal and Pelvic Floor Diseases, The Sixth Affiliated Hospital, Sun Yat-Sen University, Guangzhou, 510655 China; 4 Department of Clinical Laboratory, The Sixth Affiliated Hospital, Sun Yat-Sen University, 510655 Guangzhou, China; 5 Department of Thyroid Hernia Surgery, The Second Affiliated Hospital of Guangzhou Medical University, Guangzhou, 510260 Guangdong China

**Keywords:** LAD1, Gastric cancer, Vimentin, MAEA, Ubiquitination

## Abstract

**Background:**

Ladinin-1 (LAD1), an anchoring filament protein, has been associated with several cancer types, including cancers of the colon, lungs, and breast. However, it is still unclear how and why LAD1 causes gastric cancer (GC).

**Methods:**

Multiple in vitro and in vivo, functional gains and loss experiments were carried out in the current study to confirm the function of LAD1. Mass spectrometry was used to find the proteins that interact with LAD1. Immunoprecipitation analyses revealed the mechanism of LAD1 involved in promoting aggressiveness.

**Results:**

The results revealed that the LAD1 was overexpressed in GC tissues, and participants with increased LAD1 expression exhibited poorer disease-free survival (DFS) and overall survival (OS). Functionally, LAD1 promotes cellular invasion, migration, proliferation, and chemoresistance in vivo and in vitro in the subcutaneous patient-and cell-derived xenograft (PDX and CDX) tumor models. Mechanistically, LAD1 competitively bound to Vimentin, preventing it from interacting with the E3 ubiquitin ligase macrophage erythroblast attacher (MAEA), which led to a reduction in K48-linked ubiquitination of Vimentin and an increase in Vimentin protein levels in GC cells.

**Conclusions:**

In conclusion, the current investigation indicated that LAD1 has been predicted as a possible prognostic biomarker and therapeutic target for GC due to its ability to suppress Vimentin–MAEA interaction.

**Supplementary Information:**

The online version contains supplementary material available at 10.1186/s12967-023-04401-2.

## Introduction

Gastric cancer (GC) is the fifth most common cancer in the world and the third main cause of cancer-related deaths [1]. The prognosis for GC has substantially improved as a result of the emergence of numerous therapy options [2, 3], although the principal therapies are still radical surgery and chemotherapy. Cancer recurrence and treatment resistance continue to be among the leading causes of death in GC patients. Understanding the mechanism of chemoresistance is critically necessary to provide novel treatment targets.

As a member of the ankyrin family and a constituent of the dermal-epidermal junction membrane, LAD1 is a gene that encodes a collagen-anchored silk protein of the basal layer [4–7]. It can be found in the cell membrane, cytoplasm, and cytoskeleton and has been detected in various tissues of the gastrointestinal tract, kidney, prostate, placenta, and hematopoietic stem cells [8–10]. LAD1 gene variants have been associated with linear IgA disease, an autoimmune vesicular disease [11]. It can form the cytoskeleton of breast cancer by interacting with actin cross-linking proteins and mediating cancer cell migration and proliferation via the EGF/ERK pathway [10]. Furthermore, it is highly expressed in colorectal cancer metastatic tissues, and its expression is closely associated with prognosis, proliferation, and metastasis [8]. LAD1 may promote tumor cell malignancy and play an important role in tumor onset, development, and persistence when present.

Vimentin is a type III intermediate filament protein that is essential for the epithelial-mesenchymal transition (EMT). When the expression of Vimentin is increased and the expression of E-cadherin is decreased in epithelial cells, cells undergo EMT [12]. Vimentin helps tumor cells break out from carcinoma in situ and invade blood or lymph arteries, thereby providing them with the potential for proliferation [13, 14]. Extensive study has proven an association between vimentin and tumor invasion and metastasis [15]. Furthermore, research has shown that tumors acquire drug resistance by acquiring tumor cell stemness during the EMT process [4, 16, 17]. As a result, Vimentin protein therapy could become a viable option for the treatment of cancerous growths.

The activity of the ubiquitination system is regulated by the E3 ubiquitin ligases, which are responsible for the precise identification of target proteins [18, 19]. A wide range of developmental abnormalities, malignancies, and neurological diseases may result from defective ubiquitination, which is frequently caused by mutations in the genes that make E3 ubiquitin ligases or deubiquitinases or incorrect expression [20]. Considering that ubiquitinates are responsible for cancer, neurological disorders, and developmental difficulties, regulating their activity may provide possibilities for therapy [21]. The E3 ubiquitin ligase MAEA (macrophage erythroblast attacher, E3 Ubiquitin Ligase) functions to suppress cytokine receptor signaling through autophagy and maintain the function of hematopoietic stem cells. In vivo, studies in mice have shown that MAEA is produced by macrophages but not erythroblasts, with the latter assisting to maintain the erythroblast islands in adult mice’s bone marrow [22]. Therefore, we proposed that MAEA plays a crucial part in regulating cell stemness. Its importance in malignancies is yet unknown, and further research is needed to fully understand its function in tumor cell stemness.

In the current work, we explored the relationship between LAD1 expression and clinicopathological characteristics as well as overall survival (OS) in GC. Additionally, we used in vitro analysis to examine its molecular function in the development of cancer. LAD1 has the potential to be used as a therapeutic target and prognostic biomarker in GC since it inhibits the interaction between Vimentin and MAEA.

## Materials and methods

### Patients and tissue samples

We acquired 168 primary cancer tissue samples from Sun Yat-Sen University, Sixth Affiliated Hospital in Guangzhou, China, between December 2007 and March 2012, for our earlier studies [23–25]. The same results and clinicopathological features were obtained when we used these samples to construct tissue microarrays (TMAs) for immunohistochemistry (IHC) analysis. To examine the expression of the LAD1 protein, we also acquired eight matched sets of fresh GCs and their neighboring normal tissues.

### Immunohistochemistry

IHC staining was processed by the biotin-streptavidin horseradish peroxidase (HRP) detection system (ZSGB Bio, China) as described in a previous investigation [23]. Antibodies used in IHC were as followed: Vimentin (10366-1-AP, Proteintech, China, 400), MAEA (28363-1-AP, Proteintech, China, 1:400), and LAD1 (16136-1-AP, Proteintech, China, 1:800).

### Cell lines and culture

Similar to our previous study, the Type Culture Collection Cell Bank of the Chinese Academy of Sciences Committee (Shanghai, China) supplied four cell lines (HGC27, AGS, MKN45, NUGC3) and one human normal gastric mucosal cell (GES1). The RPMI 1640 (Corning, USA) medium was used for the cultivation of all cell lines. 10% fetal bovine (Gibco, USA) serum was added to every medium. Humidified air containing 5% CO_2_ was utilized for growing the cells at 37 °C.

### Wound healing, invasion, and migration assay

A total of 4 × 10^4^ cells were spread in the cell scratch experiment mold of a 12-well plate. After 12 h of culture, we removed the mold and added an RPMI-1640 medium containing 1% FBS. After that, we used Incucyte Zoom to take pictures of the scratch area every hour to track cell migration and scratch closure. The migration and invasion assays were carried out following our prior study’s protocol, whereas the wound healing assay was carried out in a similar way [23].

### Plasmid construction and transfection

The full-length open reading frame (ORF) of LAD1 (NM_005558.4) was used to generate a PCR amplicon, which was then cloned into the HA-tagged pCDH-CMV-MCS-EF1-CopGFP-T2A-Puro (PCDH) vector. Using the same strategy, MAEA (NM_001017405.3) and Vimentin (NM_003380.5) plasmids were cloned into pCDNA3.1 harboring either an MYC-tag or a Flag-tag. Plasmids of Ub-K63 and Ub-K48 with an HA-tag were purchased from Addgene. The shRNA of LAD1 was purchased from Genepharma (Shanghai, China). LAD1 target sequences included: ShRNA-1, 5-GCCTCAGAGAAGACATCTCTA-3; ShRNA-2, 5-CTTTCGGATGAAACCCAAGAAA-3. Lentivirus infection was used to generate stable cell lines, and the transient infection method was the same as in the previously reported study [23].

### RNA extraction and qRT-PCR

RNA extraction was obtained using the kits according to the instruction (EZB-RN4, EZBioscience, China), and the RNA was reversed to cDNA by using the kits (EZB-RT2GQ, EZBioscience, China). The following qRT-PCR was performed as previously reported [23, 24].

The primers used in the study were as follows: GAPDH, 5-GACAGTCAGCCGCATCTTCTT-3 (forward) and 5-AATCCGTTGACTCCGACCTTC-3 (reverse); LAD1, 5-AAAGCAGGAAAAGCGACCACT-3 (forward) and 5-CGGAGTTTATTTAGGCGCTCTT-3 (reverse); Vimentin, 5-AGTCCACTGAGTACCGGAGAC-3 (forward) and 5-CATTTCACGCATCTGGCGTTC-3 (reverse); MAEA, 5-GAGACTGGACGCTGTGAGAC-3 (forward) and 5-AGGTCCTTGTACGGGGAGATG-3 (reverse).

### Western blot analysis

The protein was extracted using RIPA buffer from Service-Bio in Wuhan, China, which also contains phosphatase and protease inhibitors, and the protein concentration was assessed using a BCA kit from the same company. The ensuing steps were the same as those in our earlier investigation. The incubated antibodies include LAD1 (16136-1-AP, Proteintech, China, 1:1000), GAPDH (60004-1-Ig, Proteintech, China, 1:1000), Vimentin (10366-1-AP, Proteintech, China, 1:1000), MAEA (28363-1-AP, Proteintech, China, 1:1000), HA-tag (66006-2-Ig, Proteintech, China, 1:1000), Flag (F1804, Sigma, China, 1:1000), 6×His (10001-0-AP, Proteintech, China, 1:1000), and Myc-tag (60003-2-Ig, Proteintech, China, 1:1000).

### Colony growth assay

For 3 days, the medium was changed after 600 cells had been seeded in a 6-well plate. Cells were seeded at a density of 2 × 10^4^ per well on a 6-well plate, and after 24 h, the medium was changed with or without Oxaliplatin (OXA, TargetMol, Shanghai, China). After being cultured for 10–14 days, the cells were fixed in 4% paraformaldehyde and stained with crystal violet. Images of the cells taken using a microscope were processed in Image J using an Olympus camera (Tokyo, Japan). For 3D colony formation, 2000 single cells were seeded in 200 µL of culture medium in an ultra-low attachment microplate (7007; Corning, USA) and cultured for 12 days, with medium changes occurring every 3 days with or without OXA. During this time, the tumor spheres were photographed every 4 h using an Incucyte Zoom, and their volumes were calculated (Volume = 4/3πR3).

### Cell proliferation assay

In a 96-well plate, 2 × 10^3^ cells were seeded, and after 24 h, the medium was changed with or without OXA, the confluence was photographed using an Incucyte Zoom every 2 to 4 h, and cell viability was determined using a cell count kit-8 (CCK8).

### Immunofluorescence (IF) assay

The protein was extracted using a low salt lysis buffer and the cells were lysed and centrifuged at 4 ℃. The cell supernatant was incubated with the antibody and beads (HY-K0202-5mL, MCE, USA) at 4 ℃ overnight. The remaining steps were reported in our previous study [23, 26]. The incubated antibodies include HA-tag (66006-2-Ig, Proteintech, China, 1:1000), Flag (F1804, Sigma, China, 1:1000), 6×His (10001-0-AP, Proteintech, China, 1:1000), and MYC-tag (60003-2-Ig, Proteintech, China, 1:1000).

### Mice experiment

Located at the Experimental Animal Center of the Sixth Affiliated Hospital at Sun Yat-sen University, the BALB/c nude mice were acquired from GEMPHARMATECH (Guangdong, China). Female BALB/c nude mice (n = 5; 4 weeks old) had 5 × 10^6^ cells (MKN45-ShNC/Sh1/Sh2) in 100 ul PBS injected subcutaneously into their left flanks. Tumor volumes were assessed every three days using the formula V = W^2^ L/2. Tumor size was evaluated four weeks after, by sacrificing the mice.

For the construction of a model of lung metastasis, we injected 100 µl of PBS containing 5 × 10^6^ MKN45-ShNC/Sh1/Sh2 cells into the tail vein. Before being subjected to euthanization, mice were kept for a total of 60 days. After that, their lungs were formalin-fixed, cut into slices, and screened for metastatic nodules.

NOD-SCID mice were acquired from GEMPHARMATECH (Guangdong, China) and kept in the Experimental Animal Center of The Sixth Affiliated Hospital, Sun Yat-sen University to establish GC PDX. The GC tissues used in this investigation were collected with permission and ethical authorization (SYSU-IACUC-2,022,051,303) from Sun Yat-sen University. In brief, 2 mm in diameter diced fresh tumor tissue was cleansed in PBS containing 1% penicillin-streptomycin before being subcutaneously implanted into NOD-SCID mice. Tumor volumes between 500 and 1500 mm3 (P0) showed successful engraftment; tumors were subsequently inserted to create P2-P6 mice for additional research. The P2 mice were randomly split into four groups (n = 5 per group) and given one of four pharmacological treatments: SiNC + PBS, LAD1-SiRNA + PBS, SiNC + OXA, or LAD1-SiRNA + OXA (i.p. injections of PBS and oxaliplatin every three days, and a dosage of 5 nmol kg1 of siRNA every three days). The siRNA was provided by Genepharma (Shanghai, China) for use in vivo. The mice were euthanized, and tumors were excised, fixed in formalin, and embedded in paraffin for IHC analysis when the tumor volume reached 1500 mm^3^ or when the entire period of treatment was complete.

### Online databases

Differential expression of LAD1 between GC and normal tissues was investigated through Oncomine (www.oncomine.org) and TIMER2.0 (http://timer.cistrome.org/), accessed on 12 June 2022.

### Statistical analysis

Both GraphPad Prism 7.0 (San Diego, CA, USA) and IBM SPSS Version 21.0 (IBM, New York, NY, USA) were employed for analyzing the data. The means and standard deviations of continuous data were examined for statistical significance using the student’s t-test. Depending on the characteristics of the categorical variable, the Chi-square test or Wilcoxon signed-rank test was used to determine statistical significance. The log-rank test has been used for statistical analysis, and the Kaplan-Meier method for calculating survival was also included. Using a backward-elimination method, Cox proportional hazards regression was used in a multivariate study to assess the importance of putative prognostic factors for survival.

## Results

### The LAD1 expression level was upregulated in GC patients samples

**Fig. 1:**
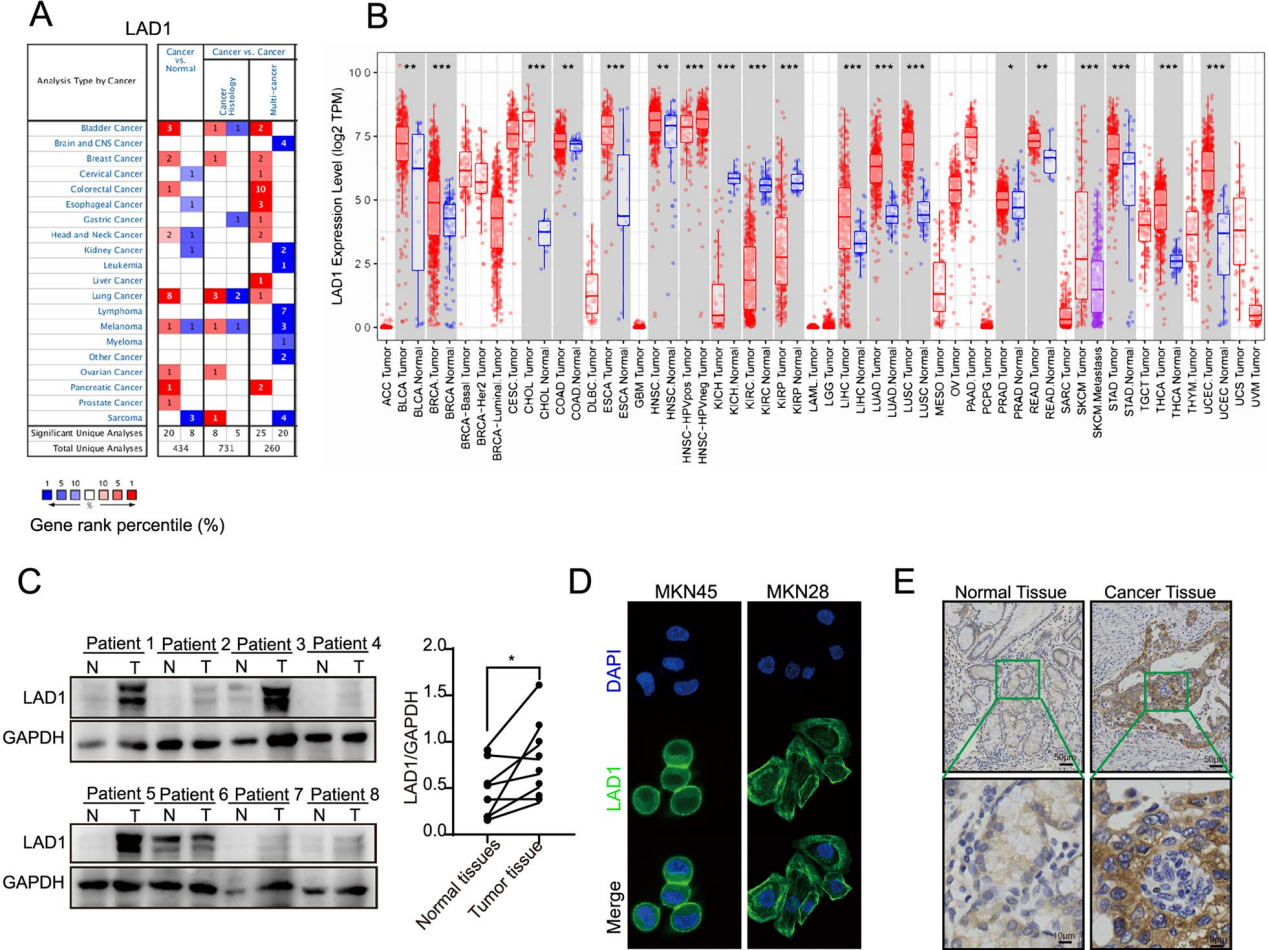
The LAD1 expression level was upregulated in GC patients samples. **A**, **B** LAD1 expression in different tumors and normal tissues were analyzed via the Oncomine database. **C** LAD1 protein expression in GC tissues was assessed through representative western blotting. **D**, **E** Endogenous LAD1 location in GC cells and tissues was determined through immunofluorescence and immunohistochemistry staining. Significance: *p < 0.05, **p < 0.01, ***p < 0.001

Data from the online public databases Oncomine and TIMER2 showed that LAD1 was upregulated more in GC than in normal tissues (Fig. 1A, B). The results from western blotting showed that gastric tissues had higher levels of LAD1 protein than normal tissues (Fig. 1C).

Through the utilization of IF and IHC tests, the results suggested that LAD1 was localized in the cytoplasm and membrane (Fig. 1D, E). These findings revealed that the expression of LAD1 was upregulated in GC.

### Increased LAD1 expression indicated a poor GC prognosis

**Fig. 2:**
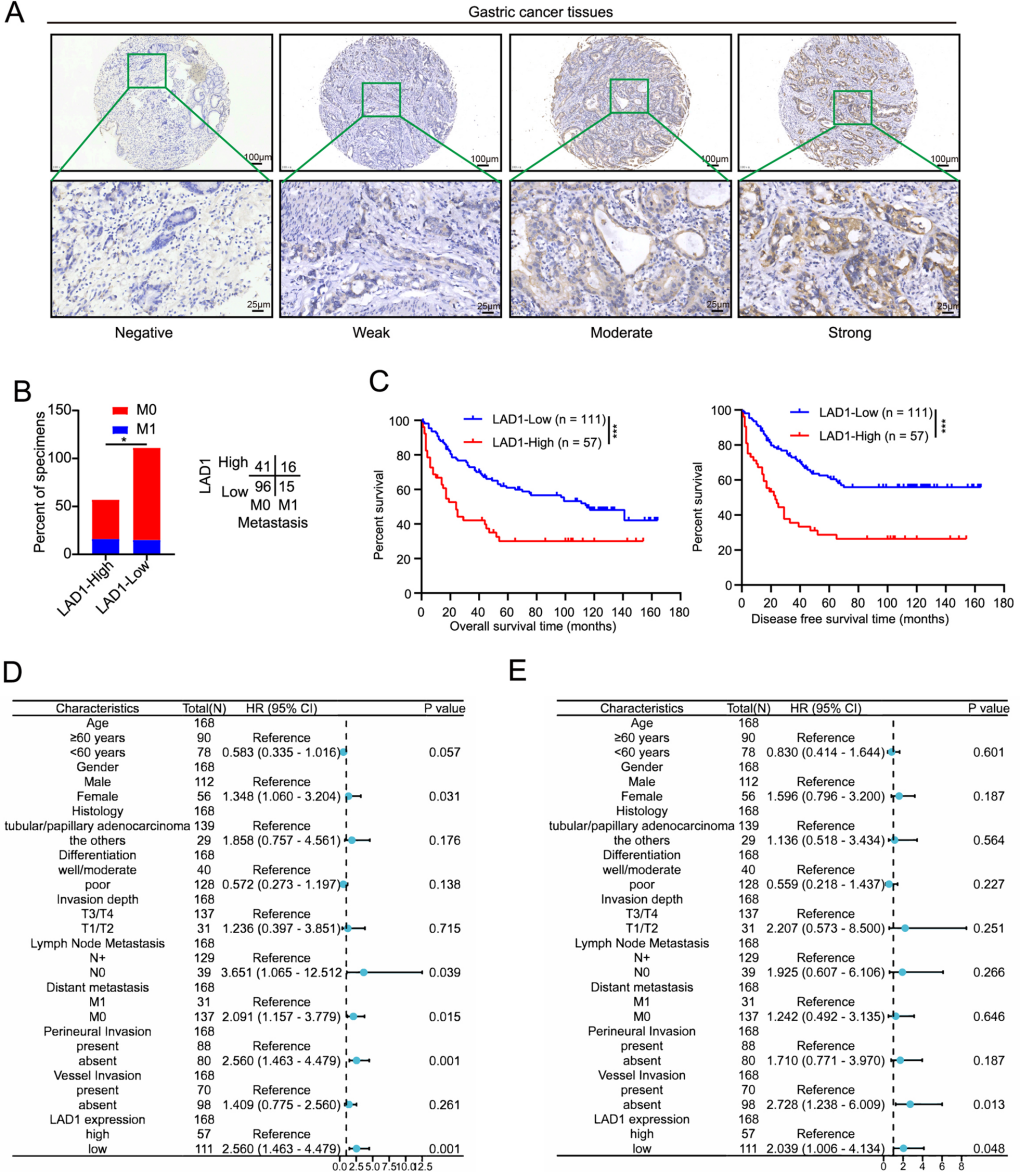
Increased LAD1 expression indicated a poor GC prognosis. **A** Representative findings of LAD1 protein expression through IHC, which were graded based on staining intensity in 168 GC tissues. **B** IHC findings show the correlation between metastasis status and LAD1 expression. **C** OS and DFS according to LAD1 expression. **D-E** Forest plots of multivariate Cox regression analyses showing the significance of different prognostic variables in GC OS and DFS. Significance as above

In contrast to low LAD1 expression, high LAD1 expression was associated with metastasis and a shorter OS and DFS (Fig. 2B, C, respectively). According to our research, high LAD1 expression was also associated with a worse prognosis in colorectal, liver, esophageal, and pancreatic malignancies (Additional file 1: Fig. S1). Furthermore, multivariate analysis revealed that LAD1 expression is an independent predictor of OS and DFS (Fig. 2D, E). These results suggest that LAD1 may play a promoter role in GC.

### LAD1 enhances cell proliferation and chemoresistance in vitro

**Fig. 3:**
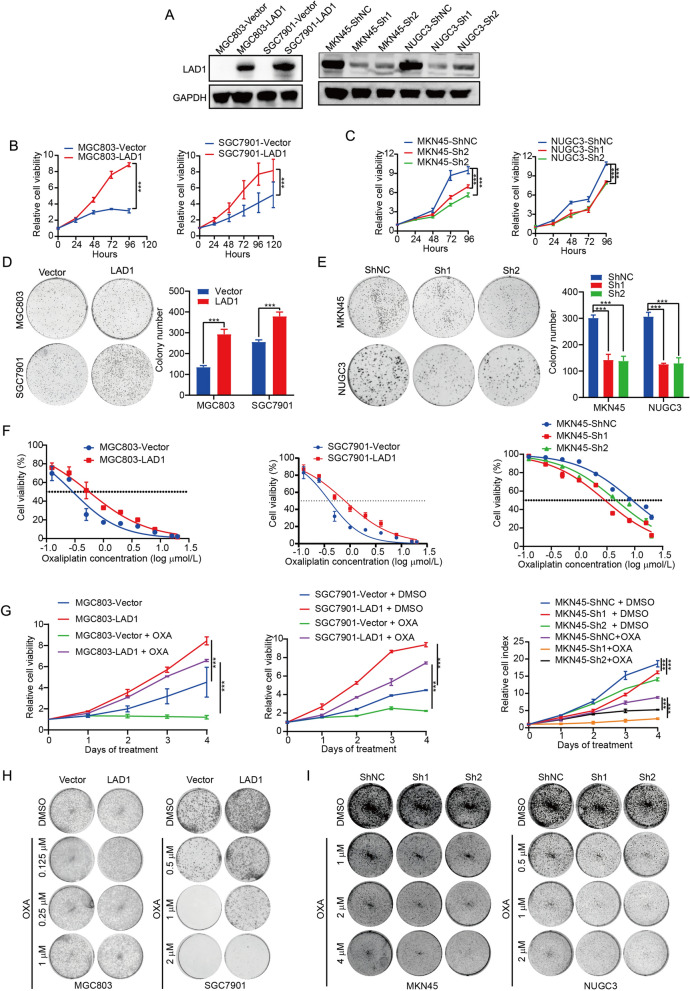
LAD1 enhances cell proliferation and chemoresistance in vitro. **A** Western blotting of LAD1 in GC cells transfected with ShLAD1 and LAD1. **B**, **C** The effect of ShLAD1 and LAD1 on the viability of the specified GC cells that were transfected. **D**, **E** Colony-forming potential of ShLAD1 and LAD1 transfected GC cells. **F** Cell survival after being exposed to various OXA concentrations over 96 h. **G** The growth curves of the indicated cells treated with OXA. **H**, **I** Colony-forming potential of indicated cells treated with different OXA concentrations

Stable cell lines (Fig. 3A) were developed and several in vitro experiments were carried out to study the role of LAD1 in gastric carcinogenesis. Our findings demonstrated that overexpression of LAD1 increased the capacity for colony formation (Fig. 3D) and cellular proliferation (Fig. 3B), and vice versa (Fig. 3C, E). Our data showed that overexpression of LAD1 significantly decreased cell death induced by oxaliplatin (Fig. 3F), as measured by cell proliferation (Fig. 3H) and colony formation (Fig. 3J). Contrarily, when oxaliplatin was used, the knockdown of LAD1 had the opposite result from the overexpression of LAD1 (Fig. 3F–I). According to these results, LAD1 increases cell proliferation while lowering oxaliplatin-induced cell death, increasing chemoresistance.

### LAD1 enhances cell invasion and migration in vivo and in vitro

**Fig. 4:**
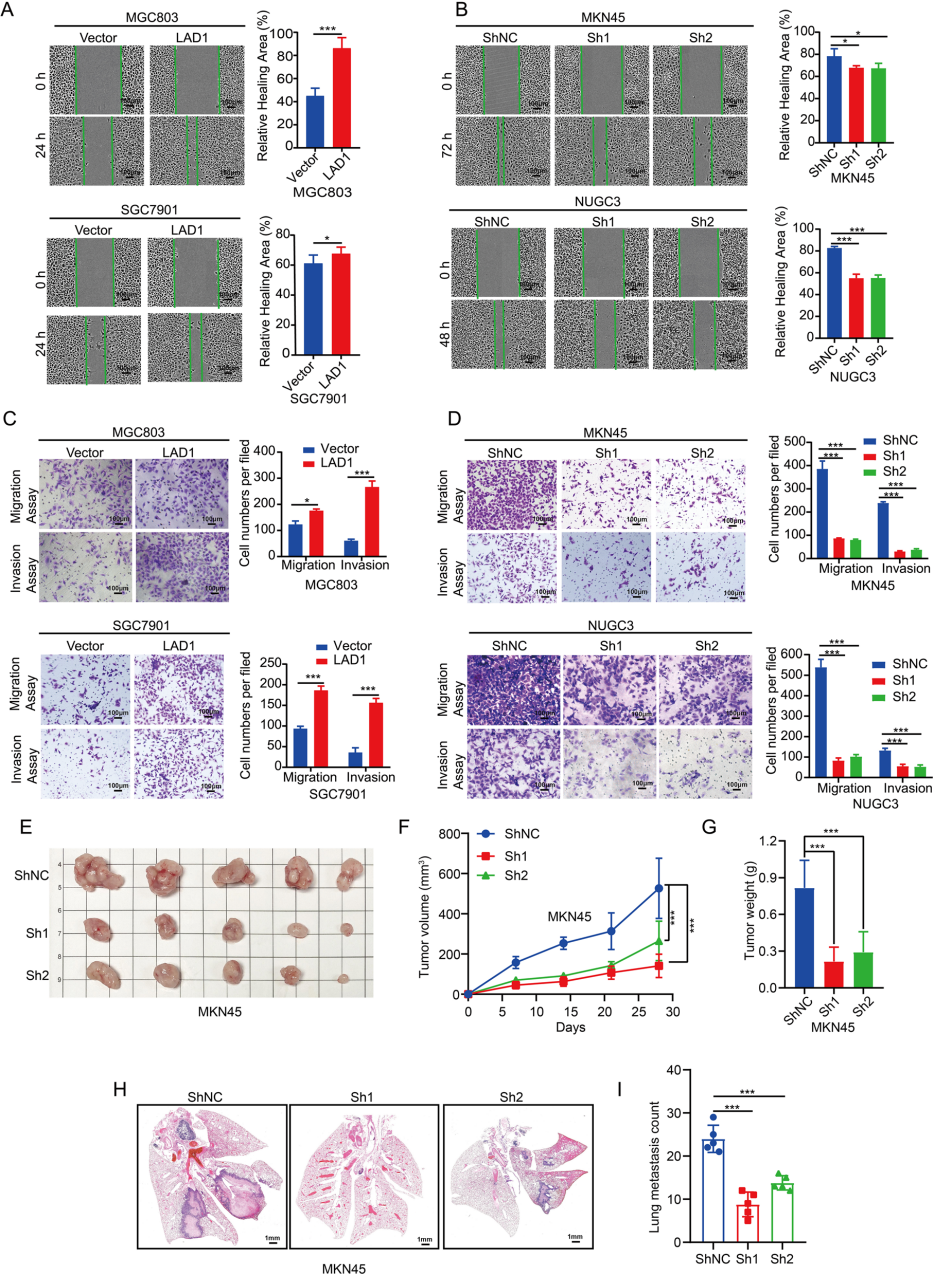
LAD1 enhances cell invasion and migration in vivo and in vitro. **A**, **B** Wound healing ability when transfected with ShLAD1 and LAD1. **C**, **D** Invasion and migration ability when transfected with ShLAD1 and LAD1. **E** Xenograft tumor clustering and overall state in MKN45 cells after LAD1 knockdown (n = 5 in every group). **F** Tumor volume growth curve in three groups. **G** Tumor weight of xenografts in the three groups. **H** Lung metastasis in MKN45 cells with LAD1 knockdown. **I** Lung metastasis count in the three groups. Significance as above

The prognosis and therapeutic options for GC are severely constrained by metastasis, which involves both cell invasion and migration. We investigated the role of LAD1 in cell migration and invasion using transwell assays and wound healing after finding that LAD1 was significantly linked with GC metastases. Overexpression of LAD1 strengthens the wound healing ability in MGC803 and SGC7901 cells (Fig. 4A), and vice versa in MKN45 and NUGC3 cells (Fig. 4B). Overexpression of LAD1 consistently increased cell invasion and migration in MGC803 and SGC7901 cells (Fig. 4C), whereas it had the reverse effect in MKN45 and NUGC3 cells (Fig. 4D).

Following that, we established a xenograft tumor model to investigate the role of LAD1 in tumor growth. When compared to the control, LAD1 knockdown results in reduced tumor growth, including weight and volume (Fig. 4E–G). We additionally developed lung metastasis via tail injection to test the effect of LAD1. LAD1 knockdown, as expected, may reduce lung metastases (Fig. 4H, I). These findings imply that LAD1 increases cellular invasion and migration both in vivo and in vitro.

### LAD1 promotes vimentin stabilization by weakening its ubiquitination

**Fig. 5:**
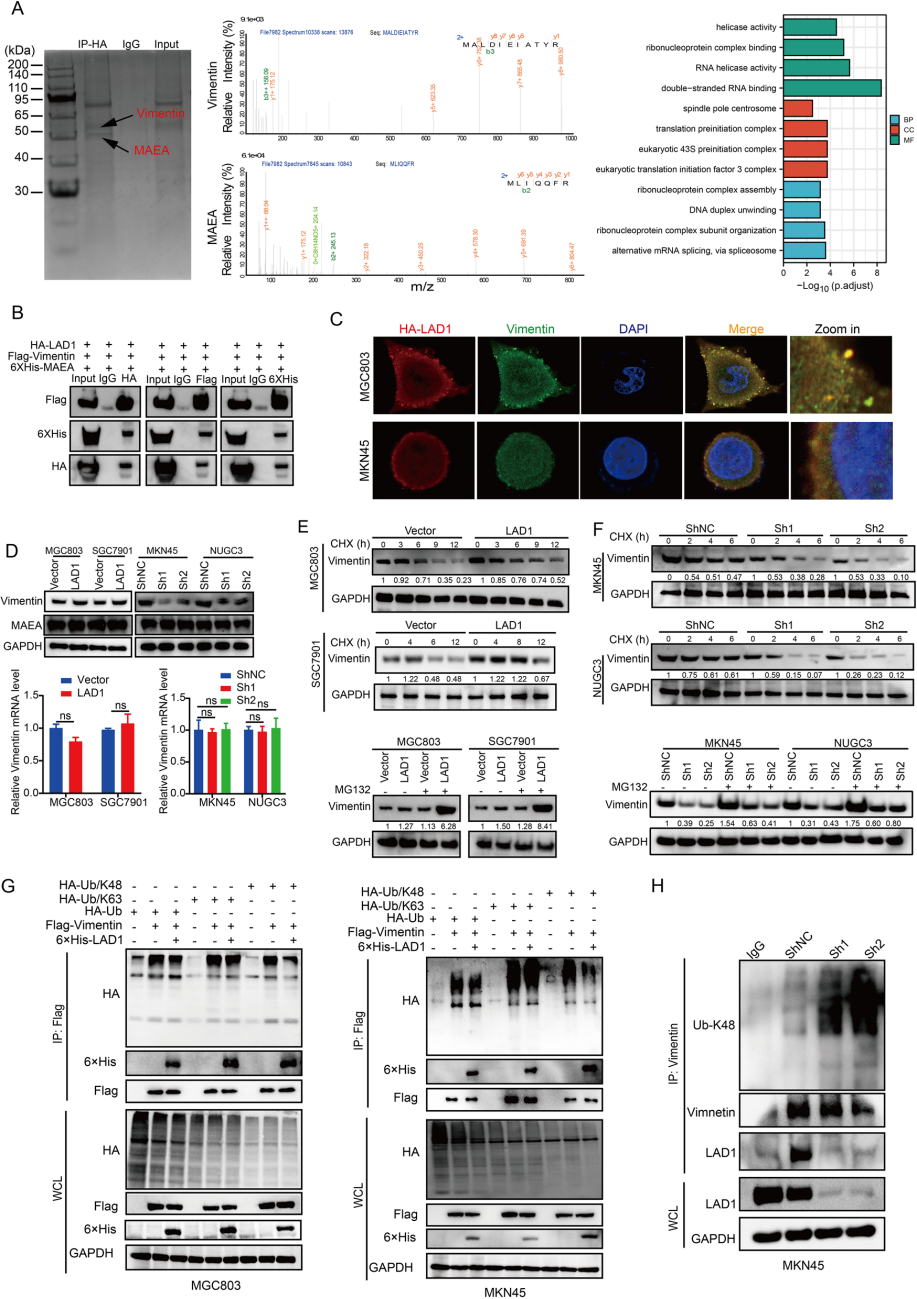
LAD1 promotes Vimentin stabilization by weakening its ubiquitination. **A** Proteins immunoprecipitated with HA were separated by SDS-PAGE from MGC803 cells overexpressing HA-LAD1. Bands close to 50–70 kDa were manually excised, identified by mass spectrometry, and analyzed using GO Oncology for specific proteins. **B** Co-IP using antibodies against HA, Flag, and 6xHis revealed the exogenous interaction between LAD1, Vimentin, and MAEA. **C** Representative co-staining images of LAD1 and Vimentin in MGC803 and MKN45 cells overexpressing HA-LAD1. **D** LAD1 dose-dependently increased Vimentin protein expression without affecting its mRNA expression. **E**, **F** Western blotting of Vimentin levels in GC cells with transfected LAD1 and ShLAD1 with CHX and MG132 treatment. **G** The levels of Vimentin ubiquitination were specified via Western blotting in MGC803 and MKN45 cells following transfection with the respective plasmids. **H** Western blotting of Vimentin ubiquitination in MKN45 cells following the knockdown of endogenous LAD1. Significance as above

Our results showed that Vimentin and MAEA had significant correlations with LAD1 (Fig. 5A), which was further confirmed by co-immunoprecipitation and co-staining assays (Fig. 5B, C). We discovered that LAD1 could increase Vimentin protein levels but not mRNA levels (Fig. 5D), showing that LAD1 regulates Vimentin post-transcriptionally. Overexpression of LAD1 inhibited Vimentin degradation, but knockdown of LAD1 increased Vimentin degradation considerably when treated with cycloheximide (CHX) (Fig. 5C), implying that LAD1 could prolong Vimentin half-life. Treatment of cells with the proteasome inhibitor (MG132) for 2 h increased LAD1-mediated stabilization, and this procedure was carried out to determine whether LAD1 weakens the ubiquitin-proteasome degradation of vimentin (Fig. 5E, F). Furthermore, we found that the overexpression of LAD1 could decrease the K48-linked polyubiquitination of Vimentin, resulting in increased Vimentin protein levels (Fig. 5G). In contrast, the knockdown of LAD1 failed to decrease the polyubiquitination of Vimentin (Fig. 5H), indicating that LAD1 competitively binds to Vimentin, disrupting its interaction with MAEA, an E3 ubiquitin ligase, and resulting in decreased K48-linked ubiquitination of Vimentin, leading to increased Vimentin protein levels in GC cells.

### LAD1 abrogates the Vimentin-MAEA interaction

**Fig. 6:**
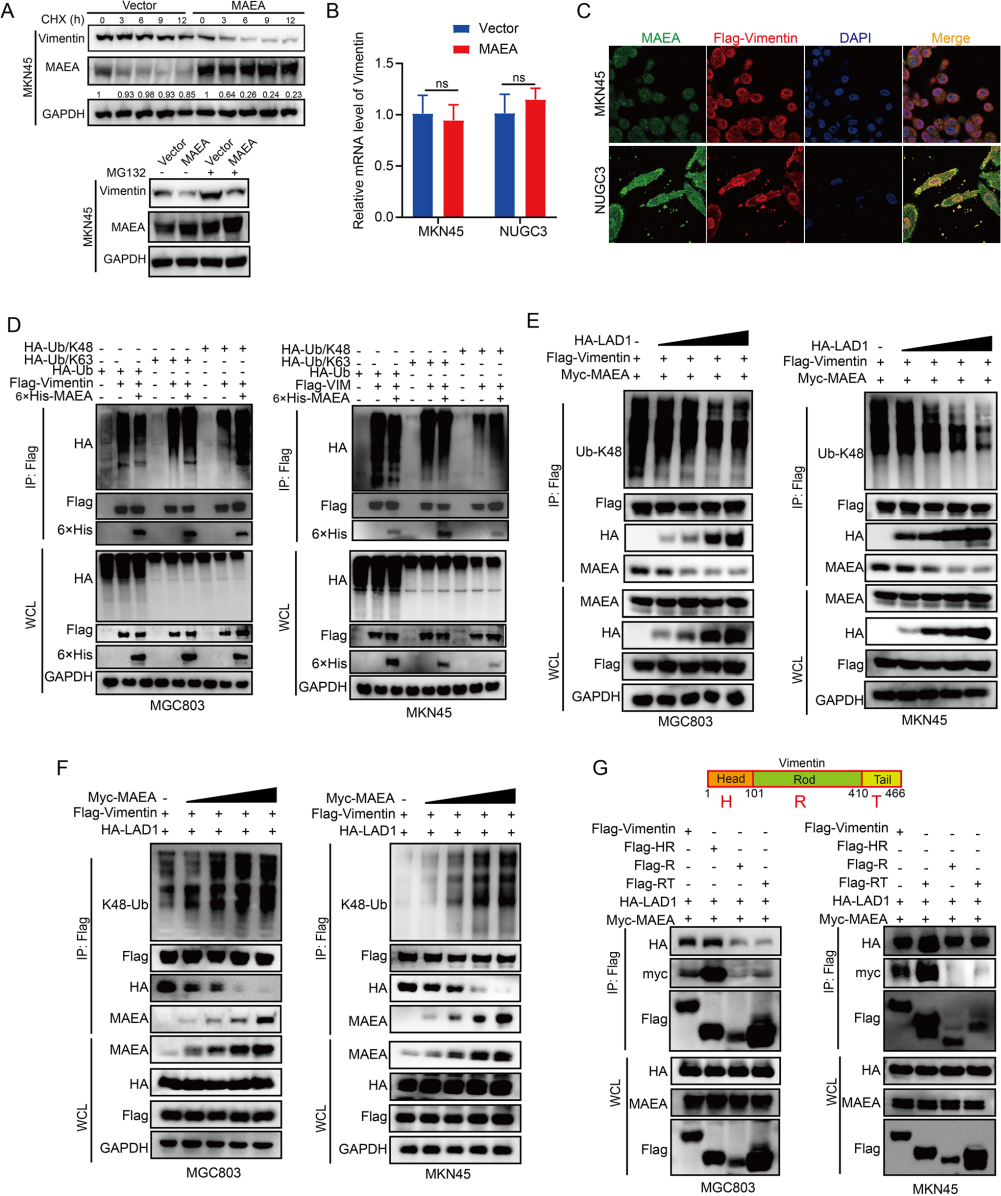
LAD1 abrogates the Vimentin-MAEA interaction. **A** Western blotting of Vimentin levels in GC cells with transfected MAEA with CHX and MG132 treatment. **B** The mRNA level of Vimentin in MKN45 and NUGC3 cells after being transfected with MAEA. **C** Representative co-staining images of MAEA and Vimentin in MGC803 and MKN45 cells overexpressing Flag-Vimentin. **D** Western blot analysis of Vimentin ubiquitin in MGC803 and MKN45 cells after transfecting the indicated plasmid. **E** LAD1 inhibited the K48-ubiquitination of Vimentin protein in a dose-dependent way. **F** MAEA promoted the K48-ubiquitination of Vimentin protein in a dose-dependent way. **G** Co-IP tests were carried out to examine the interaction between LAD1, MAEA, and several Flag-tagged Vimentin truncated mutants in MGC803 and MKN45 cells

We investigated the role of the novel E3 ligase, MAEA [22], in mediating the degradation of Vimentin and whether LAD1 could abrogate this process in GC. When treated with CHX, we discovered that MAEA overexpression greatly accelerated the degradation of vimentin (Fig. 6A), indicating that MAEA may reduce the half-life of vimentin. We exposed the cells to MG132 for two hours to examine whether MAEA was a factor in the ubiquitin-proteasome-mediated degradation of vimentin. We found that MAEA-mediated stabilization of Vimentin was weakened by MG132 (Fig. 6A). In a similar manner to LAD1, we also discovered that MAEA could lower the protein level of vimentin but not its mRNA level (Fig. 6B). In the meantime, IF staining further supported the co-localization of MAEA and Flag-Vimentin in the cytoplasm. Additionally, our research revealed that in MGC803 and MKN45 cells, MAEA could bind to Vimentin and cause K48-linked Vimentin polyubiquitination (Fig. 6D). Interestingly, we observed that LAD1 could dose-dependently decrease MAEA binding to Vimentin and boost the Vimentin protein level in both cell lines (Fig. 6E). Conversely, the addition of MAEA decreased LAD1 binding to Vimentin and reduced the Vimentin protein level in a dose-dependent manner (Fig. 6F). As shown in Fig. 6G, we also discovered that both LAD1 and MAEA bound to the Head-Rod (HR) domain of Vimentin, suggesting that LAD1 might compete with MAEA for binding to Vimentin and disrupt the interaction between Vimentin and MAEA. These findings imply that LAD1 competes with Vimentin for binding, preventing Vimentin from interacting with its E3 ligase MAEA. As a result, the level of the protein vimentin increases and K48-linked ubiquitination decreases.

#### LAD1 and vimentin were crucial for carcinogenesis in vitro and in vivo

**Fig. 7:**
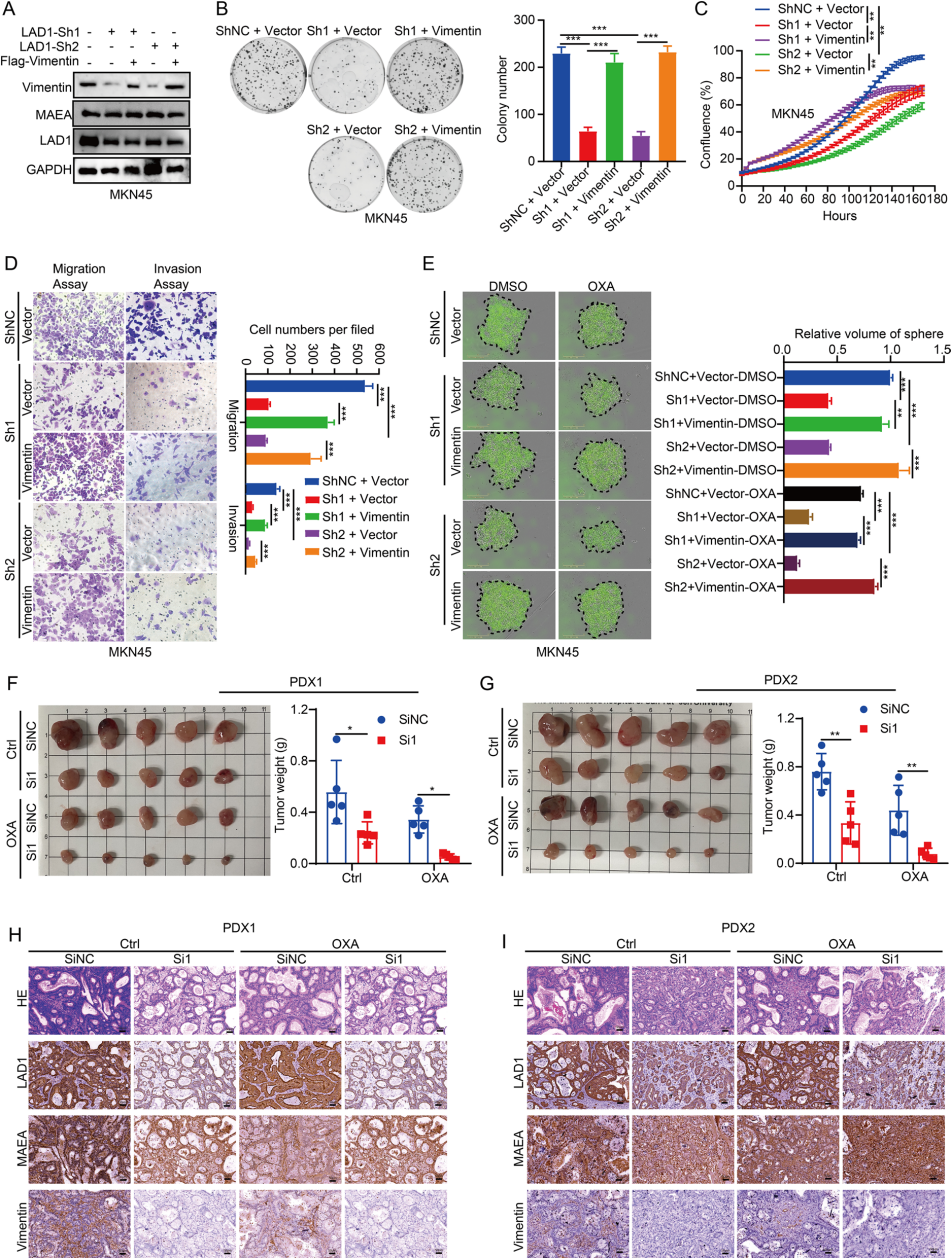
LAD1 and Vimentin were crucial for carcinogenesis in vivo and in vitro**(**cells transfected with the indicated plasmids). **A** Western blotting of MKN45 cells. **B** Analysis of colony formation ability in MKN45 cells. **C** Analysis of cell viability in MKN45 cells. **D** Quantification of invaded and migrated cells in MKN45 cells. **E** Representative images of sphere formation assays in MKN45 cells treated with or without OXA are shown. **F-G** Tumor weight of PDX model tumors after treatment with siRNA of LAD1 in vivo. **H**, **I** H&E, LAD1, MAEA, and Vimentin staining in the PDX tumor model mentioned above. Significance as above

**Fig. 8:**
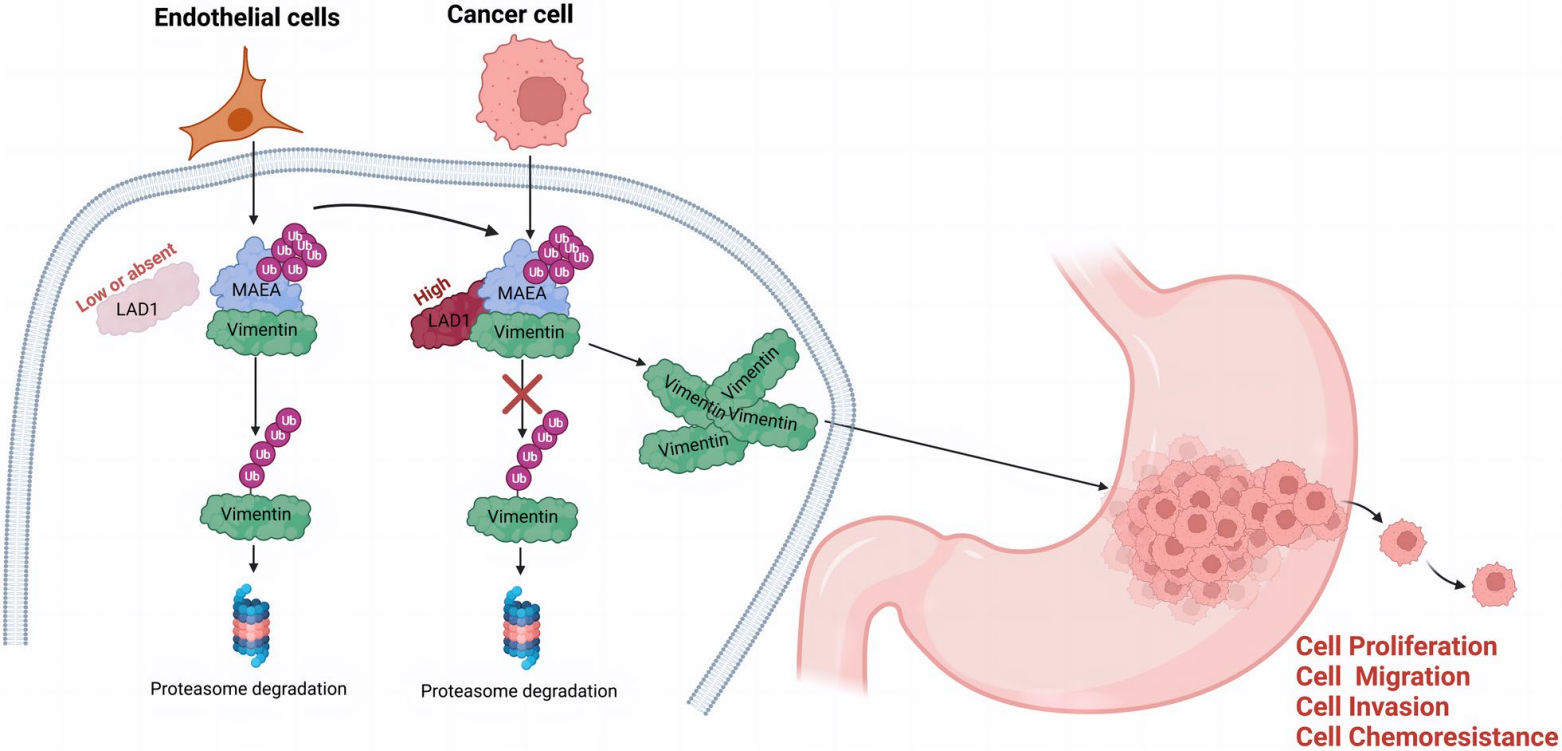
Molecular mechanism diagram depicting the role of LAD1 in regulating malignant progression of GC (Created using BioRender).

We discovered that the negative effects of LAD1 knockdown on cell migration, proliferation, invasion, and chemoresistance were substantially mitigated by the overexpression of vimentin (Fig. 7A–E). Importantly, we evaluated the potential therapeutic impact of LAD1 targeting utilizing intraperitoneal siRNA and oxaliplatin injection using two PDX models. The Si1 group had reduced tumor development (weight and size) as compared to controls, particularly following oxaliplatin treatment (Fig. 7F, G), showing enhanced sensitivity to oxaliplatin. Therefore, according to our research, LAD1 regulates the expression of Vimentin to support GC cell proliferation, migration, invasion, and chemoresistance (Fig. 8).

## Discussion

According to the results of the current investigation, LAD1 was shown to be strongly expressed in gastric cancer and to be significantly correlated with metastasis. Patients with high LAD1 expression in gastric cancer had worse prognoses than those with low expression. Additionally, we discovered that LAD1 can promote chemoresistance in gastric cancer in vitro and in vivo, LAD1 targeting can promote chemosensitivity, and LAD1 can stabilize Vimentin by reducing MAEA-mediated ubiquitination.

The primary type of treatment for advanced GC is chemotherapy, and the standard first-line chemotherapy regimen includes oxaliplatin [27, 28]. Chemotherapy loses its efficacy as metastases develop and cases develop resistance to chemotherapy. Depletion of LAD1 prevents colorectal cancer cells from migrating to the liver in vivo, and increased LAD1 expression is linked to metastatic colorectal cancer tissues [8]. LAD1, a novel protein, has been implicated in the development of cancer. In the meantime, LAD1 knockdown in an animal model inhibited the expression of genes essential for cell survival, slowing the growth of mammary xenografts [10]. Despite its potential use as a therapeutic target and predictive biomarker in GC, the role of LAD1 in GC carcinogenesis is unknown. The new protein LAD1 considerably stabilized Vimentin by preventing MAEA-mediated ubiquitination and enhancing cellular chemoresistance, invasion, migration, and proliferation. We also discovered that high levels of LAD1 expression were associated with a poor prognosis and metastases in GC.

We analyzed the Oncomine and TCGA data to learn more about LAD1 in GC. Our results show that the mRNA level of LAD1 is considerably higher in GC than in normal tissues. We also found a strong correlation between LAD1 and metastasis, and that increased LAD1 expression was connected to poor DFS and OS among individuals with GC. Furthermore, the pan-cancer analysis revealed that LAD1 is associated with a worse outcome and accelerates carcinogenesis. Our in vitro and in vivo studies, which are comparable to those on breast and colorectal malignancies [8, 10] provide additional evidence that LAD1 increases cellular invasion and migration, chemoresistance, tumor growth, and lung metastasis in GC.

The protein vimentin has been linked to increasing GC formation and prognosis [14, 29]. Ubiquitination is the primary mechanism for Vimentin degradation in tumors [30, 31]. The ability of tumor cells to escape from neoplasia in situ tissue, infiltrate lymphatic or blood vessels, and spread to distant sites is assisted by the upregulation of Vimentin expression, which gives tumor cells the shape and properties of mesenchymal cells. Several investigations have linked vimentin to tumor invasion and metastasis. Moreover, drug resistance is thought to be acquired by tumors through the EMT process, according to some investigations [4, 16]. An E3 ligase is an essential enzyme in the ubiquitination protease degradation pathway by interacting with the target protein. MAEA is a new E3 ligase that promotes autophagy and the preservation of hematopoietic stem cells [22]. As a result, MAEA is critical in modulating cell stemness. However, its significance and function in malignant growths remain mostly unknown, and more research into its role in tumor cell stemness is required.

Mass spectrometry was used to learn more about how LAD1 contributes to carcinogenesis. Surprisingly, we found that LAD1 stabilizes vimentin rather than promoting its degradation, in addition to interacting with vimentin and the MAEA protein. An earlier investigation suggested that MAEA might be a novel E3 ligase [22]. Accordingly, we hypothesized that LAD1 served as a molecular bridge between MAEA and Vimentin during the ubiquitination proteasome degradation process. We observed that both LAD1 and MAEA may impact the ubiquitination proteasome degradation of Vimentin, as was predicted. However, whereas LAD1 inhibits Vimentin K48 ubiquitination, MAEA promotes it. Furthermore, LAD1 was discovered to bind to Vimentin competitively, inhibiting the E3 ligase MAEA from binding to Vimentin. We observed that Vimentin’s Head-Rod (HR) domain was a binding site for both LAD1 and MAEA.

In summary, our study first discovered the malignant tumor-promoting role of LAD1 in gastric cancer and discovered a novel mechanism of LAD1 inhibiting the ubiquitination of Vimentin mediated by MAEA. However, there are still limitations in this study. The relationship between LAD1 and clinicopathological characteristics, as well as MAEA and Vimentin in clinical samples, still has to be further confirmed. However, to confirm the clinical transformative value of LAD1, further PDX samples or the development of organoid models are still required.

## Conclusion

In conclusion, the current investigation showed that LAD1 expression is increased in GC and that this increase is associated with clinicopathological traits and the prognosis for GC. The OS and DFS of patients with high LAD1 expression were poorer. The process of vimentin competition and disruption with its E3 ligase MAEA, which is crucial for GC carcinogenesis, is hypothesized to be facilitated by LAD1. A potential treatment target for GC as well as a predictive biomarker has been identified by the findings of our study.

### Supplementary information

**Additional file 1: Figure S1:** Specific original records of the article modifications.

## Data Availability

All data generated or analyzed during this study are included in this published article and its supplementary information files. The raw data are available from the corresponding author upon reasonable request.

## Abbreviations

LAD1: Ladin-1
MAEA: Macrophage erythroblast attacher, E3 ubiquitin ligase
GC: Gastric cancer
OS: Overall survival
DFS: Disease-free survival
OXA: Oxaliplatin
NOD-SCID: Nonobese diabetic severe combined immunodeficiency
IF: Immunofluorescence
Co-IP: Co-immunoprecipitation
siRNA: Small interfering RNA
IHC: Immunohistochemical staining
TMAs: Tissue microarrays
HRP: Horseradish peroxidase
ORF: Open reading frame

## References

[1] Smyth EC, Nilsson M, Grabsch HI, van Grieken NC, Lordick F (2020). Gastric cancer. Lancet (London England).

[2] Sexton RE, Al Hallak MN, Diab M, Azmi AS (2020). Gastric cancer: a comprehensive review of current and future treatment. Cancer Metastasis Rev.

[3] Joshi SS, Badgwell BD (2021). Current treatment and recent progress in gastric cancer. CA Cancer J Clin.

[4] Shibue T, Weinberg RA (2017). EMT, CSCs, and drug resistance: the mechanistic link and clinical implications. Nat Rev Clin Oncol.

[5] Hogg N, Bates PA (2000). Genetic analysis of integrin function in man: LAD-1 and other syndromes. Matrix Biol.

[6] Azevedo L, Serrano C, Amorim A, Cooper DN (2015). Trans-species polymorphism in humans and the great apes is generally maintained by balancing selection that modulates the host immune response. Hum Genomics.

[7] Nawaz Tipu H, Raza R, Jaffar S, Khan A, Anwar MZ, Ahmad W (2020). β2 Integrin Gene (ITGB2) mutation spectra in Pakistani families with leukocyte adhesion deficiency type 1 (LAD1). Immunobiology..

[8] Moon B, Yang SJ, Park SM, Lee SH, Song KS, Jeong EJ (2020). LAD1 expression is associated with the metastatic potential of colorectal cancer cells. BMC Cancer.

[9] Teixeira JC, de Filippo C, Weihmann A, Meneu JR, Racimo F, Dannemann M (2015). Long-term balancing selection in *LAD1* maintains a missense trans-species polymorphism in humans, chimpanzees, and bonobos. Mol Biol Evol.

[10] Roth L, Srivastava S, Lindzen M, Sas-Chen A, Sheffer M, Lauriola M (2018). SILAC identifies LAD1 as a filamin-binding regulator of actin dynamics in response to EGF and a marker of aggressive breast tumors. Sci Signal.

[11] Leverkus M, Georgi M, Nie ZX, Hashimoto T, Bröcker EB, Zillikens D (2002). Cicatricial pemphigoid with circulating IgA and IgG autoantibodies to the central portion of the BP180 ectodomain: beneficial effect of adjuvant therapy with high-dose intravenous immunoglobulin. J Am Acad Dermatol.

[12] Ridge KM, Eriksson JE, Pekny M, Goldman RD (2022). Roles of vimentin in health and disease. Genes Dev.

[13] Dongre A, Weinberg RA (2019). New insights into the mechanisms of epithelial-mesenchymal transition and implications for cancer. Nat Rev Mol Cell Biol.

[14] Zhu ZL, Rong ZY, Luo Z, Yu ZL, Zhang J, Qiu ZJ (2019). Circular RNA circNHSL1 promotes gastric cancer progression through the miR-1306-3p/SIX1/vimentin axis. Mol Cancer.

[15] Lin ZJ, Wu YL, Xu YT, Li GQ, Li ZH, Liu T (2022). Mesenchymal stem cell-derived exosomes in cancer therapy resistance: recent advances and therapeutic potential. Mol Cancer.

[16] Pan GT, Liu YH, Shang LR, Zhou FY, Yang SL (2021). EMT-associated microRNAs and their roles in cancer stemness and drug resistance. Cancer Commun (Lond).

[17] Fischer KR, Durrans A, Lee S, Sheng JT, Li FH, Wong STC (2015). Epithelial-to-mesenchymal transition is not required for lung metastasis but contributes to chemoresistance. Nature.

[18] Sosič I, Bricelj A, Steinebach C (2022). E3 ligase ligand chemistries: from building blocks to protein degraders. Chem Soc Rev.

[19] Zheng N, Shabek N (2017). Ubiquitin ligases: structure, function, and regulation. Annu Rev Biochem.

[20] Rape M (2018). Ubiquitylation at the crossroads of development and disease. Nat Rev Mol Cell Biol.

[21] Popovic D, Vucic D, Dikic I (2014). Ubiquitination in disease pathogenesis and treatment. Nat Med.

[22] Wei QZ, Pinho S, Dong SX, Pierce H, Li HH, Nakahara F (2021). MAEA is an E3 ubiquitin ligase promoting autophagy and maintenance of haematopoietic stem cells. Nat Commun.

[23] Jiang YM, Yu XH, Zhao YD, Huang JT, Li TY, Chen H (2021). ADAMTS19 suppresses cell migration and invasion by targeting S100A16 via the NF-κB pathway in human gastric cancer. Biomolecules.

[24] Huang JT, Sun Y, Chen H, Liao Y, Li SM, Chen CY (2019). ADAMTS5 acts as a tumor suppressor by inhibiting migration, invasion and angiogenesis in human gastric cancer. Gastric Cancer.

[25] Huang JT, Bai Y, Huo LJ, Xiao J, Fan XJ, Yang ZH (2015). Upregulation of a disintegrin and metalloprotease 8 is associated with progression and prognosis of patients with gastric cancer. Transl Res.

[26] Li TY, Zhou JY, Jiang YM, Zhao YD, Huang JT, Li WY (2022). The novel protein ADAMTS16 promotes gastric carcinogenesis by targeting IFI27 through the NF-κb signaling pathway. Int J Mol Sci.

[27] Lordick F, Carneiro F, Cascinu S, Fleitas T, Haustermans K, Piessen G (2022). Gastric cancer: ESMO Clinical Practice Guideline for diagnosis, treatment and follow-up. Ann Oncol.

[28] Ajani JA, D’Amico TA, Bentrem DJ, Chao J, Cooke D, Corvera C (2022). Gastric cancer, version 2.2022, NCCN clinical practice guidelines in oncology. J Natl Compr Canc Netw.

[29] Wei C, Yang CG, Wang SY, Shi DD, Zhang CX, Lin XB (2019). Crosstalk between cancer cells and tumor associated macrophages is required for mesenchymal circulating tumor cell-mediated colorectal cancer metastasis. Mol Cancer.

[30] Pang K, Park J, Ahn SG, Lee J, Park Y, Ooshima A (2019). RNF208, an estrogen-inducible E3 ligase, targets soluble vimentin to suppress metastasis in triple-negative breast cancers. Nat Commun.

[31] Li Q, Deng MS, Wang RT, Luo H, Luo YY, Zhang DD (2023). PD-L1 upregulation promotes drug-induced pulmonary fibrosis by inhibiting vimentin degradation. Pharmacol Res.

