# Supplementary figures and images for: LAD1 promotes malignant progression by diminishing ubiquitin-dependent degradation of vimentin in gastric cancer
Source: J Transl Med. 2023 Sep 17;21:632. doi: 10.1186/s12967-023-04401-2 (PMC10506284; doi:10.1186/s12967-023-04401-2)

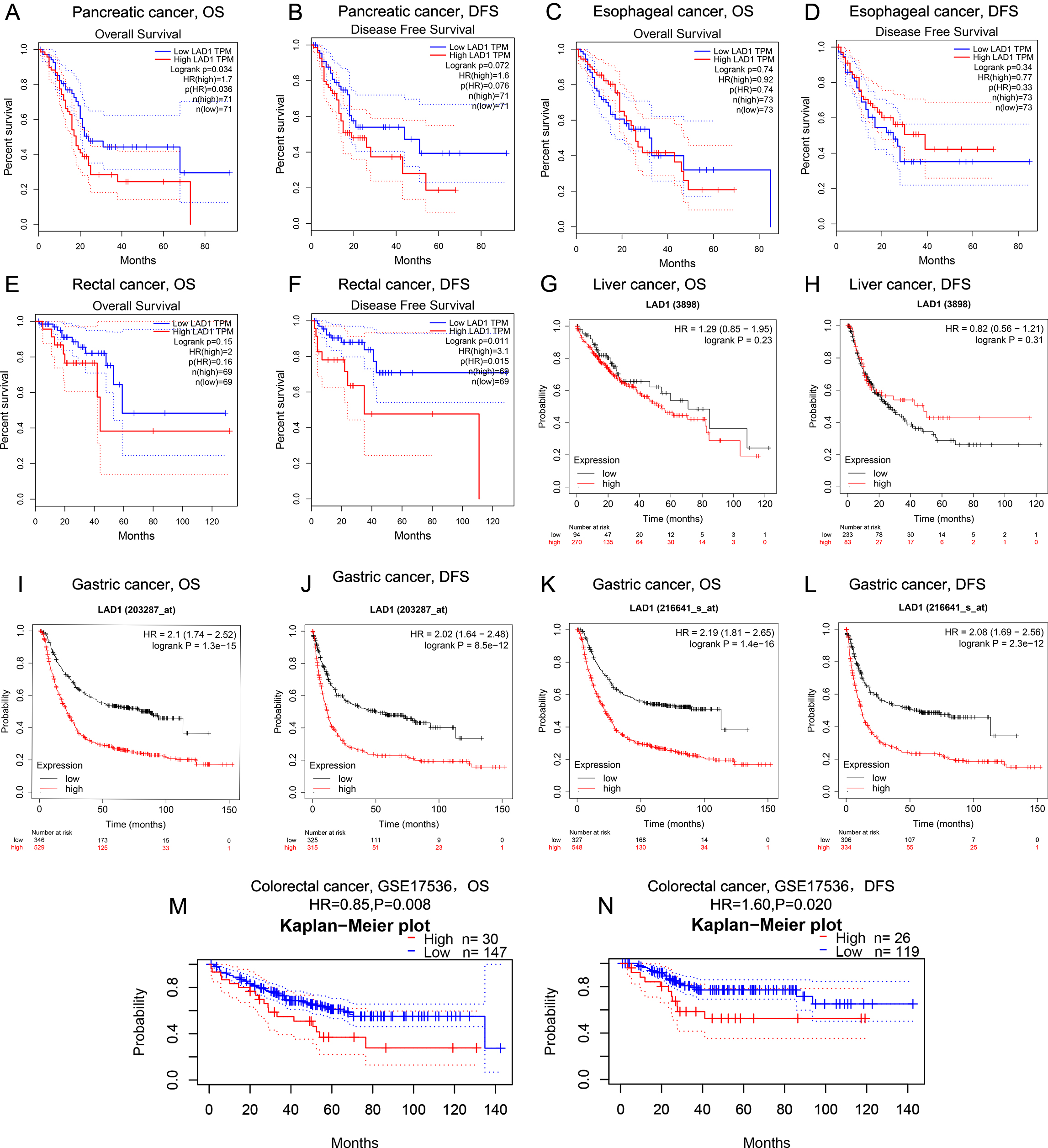

Supplement: Supplementary file 1 — Additional file 1: Figure S1: Specific original records of the article modifications. [file 12967_2023_4401_MOESM1_ESM.jpg]
